# *Salmonella* overcomes tumor immune tolerance by inhibition of tumor indoleamine 2, 3-dioxygenase 1 expression

**DOI:** 10.18632/oncotarget.6258

**Published:** 2015-10-28

**Authors:** Yu-Diao Kuan, Che-Hsin Lee

**Affiliations:** ^1^ Graduate Institute of Basic Medical Science, School of Medicine, China Medical University, Taichung, Taiwan; ^2^ Department of Microbiology, School of Medicine, China Medical University, Taichung, Taiwan

**Keywords:** salmonella, tumor-targeting, indoleamine 2, 3-dioxygenase 1, immune tolerance

## Abstract

Over the past decades, *Salmonella* has been proven capable of inhibiting tumor growth. It can specifically target tumors and due to its facultative anaerobic property, can be more penetrative than other drug therapies. However, the molecular mechanism by which *Salmonella* inhibits tumor growth is still incompletely known. The antitumor therapeutic effect mediated by *Salmonella* is associated with an inflammatory immune response at the tumor site and a T cell-dependent immune response. Many tumors have been proven to have a high expression of indoleamine 2, 3-dioxygenase 1 (IDO), which is a rate-limiting enzyme that catalyzes tryptophan to kynurenine, thus causing immune tolerance within the tumor microenvironment. With decreased expression of IDO, increased immune response can be observed, which might be helpful when developing cancer immunotherapy. The expression of IDO was decreased after tumor cells were infected with *Salmonella*. In addition, Western blot analysis showed that the expression levels of phospho-protein kinase B (P-AKT), phospho-mammalian targets of rapamycin (P-mTOR), and phospho-p70 ribosomal s6 kinase (P-p70s6K) in tumor cells were decreased after *Salmonella* infection. In conclusion, our results indicate that *Salmonella* inhibits IDO expression and plays a crucial role in anti-tumor therapy, which might be a promising strategy combined with other cancer treatments.

## INTRODUCTION

Bacterial cancer therapy can be traced back to the 19th century: a sarcoma surgeon found that bacterial infection has a beneficial influence on patients with tumors [1]. Since then, researchers have been developed many cancer therapies based on the use of bacteria, attenuated bacteria strains or bacterial products such as *Streptococcus* and *Serratia*species [2, 3], *Clostridia* species [4], and *Mycobacterium bovis*-made Bacillus Calmette-Guerin, commonly used in bladder cancer [5]. *Salmonella* is one bacterium that has been known for years to have proven antitumor efficacy. This species has several advantages that apply to cancer therapy: because it is flagellated, *Salmonella* can penetrate deeply into tumor tissue yet viruses, drugs and antibodies cannot [6] and because it is a facultative anaerobe, *Salmonella* can colonize small metastatic and larger tumors [7]. Additionally, *Salmonella* was found to replicate much more in tumors than in normal tissue. [8, 9].

Until now, one of the barriers to curing cancer has been tumor immune tolerance, which renders host immunity unable effectively to recognize or kill tumor cells; in some situations, immune cells even undergo inactivation cell cycle arrest and apoptosis [10, 11]. Some factors have been reported that give tumor cells the ability to escape from host immunity, including interferon-γ (IFN-γ) [12], galectin [13], transforming growth factor β (TGF-β) [14], and indoleamine 2, 3-dioxygenase 1 (IDO1) [15]. In particular, IDO increase the concentration of kynurenine, leading to activation of regulatory T cells, inactivation of effective T cells and even apoptosis of immune cells [16]. There are some treatments that focus on overcoming this obstacle. 1-methyl tryptophan (1-MT) is an analog of IDO substrate that has a higher affinity and is usually used in combination with chemotherapeutic drugs [17]. 1-MT exist two isoforms, 1-methyl-D-tryptophan (D-1-MT) and 1-methyl-L-tryptophan (L-1-MT). L-1- MT is considered as a more potent IDO inhibitor, while D-1-MT was commonly chosen for clinical trial with more effective antitumor activity and superior ability of abrogating immune inhibition [18]. However, there are still some concerns about administering 1-MT [19].

Previous studies have demonstrated that *Salmonella* can decrease angiogenesis and increase infiltration of immune cells within a tumor region, ultimately leading to inhibition of tumor growth [20]. Some studies indicate that *Salmonella* activates the CD8^+^ T cell immune response to eliminate tumor cells [21]. This phenomenon can be verified in a T cell-deficient mouse, in which *Salmonella*-mediated tumor regression was inhibited, as was the infiltration of neutrophils and macrophages in the tumor region [22]. These findings suggest that *Salmonella* might mainly activate CD8^+^ T cell immunity within a tumor region. Thus, we postulate that the underlying mechanism is that *Salmonella* can break IDO-mediated immune tolerance in the tumor microenvironment.

Autophagy is a term first coined by Christian de Duve and describes a process in which cells degrade misfolded or aggregated protein or even organelles to recycle the components to help the cell overcome stress [23]. Some cancer cells are believed to have a reduced autophagic property, thereby promoting oncogenesis [24]. Moreover, our and other studies indicate that *Salmonella* can induce autophagy of immune cells and tumor cells through phospho-protein kinase B (P-AKT)/phospho-mammalian targets of the rapamycin (P-mTOR) pathway [25]. The regulation of autophagy can also affected by the upstream factor controlling IDO expression, which infers that autophagy might be related to the immune response [26]. These findings connect the relationships between *Salmonella*, cancer and host immunity, while the detailed molecular mechanism still needs further investigation. As a result, we postulate that *Salmonella*-induced autophagy is involved in the process of *Salmonella* regulating IDO to hinder tumor immune tolerance. We hope these findings can lead to a potential treatment that evokes host immunity to conquer cancer.

## RESULTS

### *Salmonella* downregulated kynurenine and enhanced the viability of T cells

**Figure 1:**
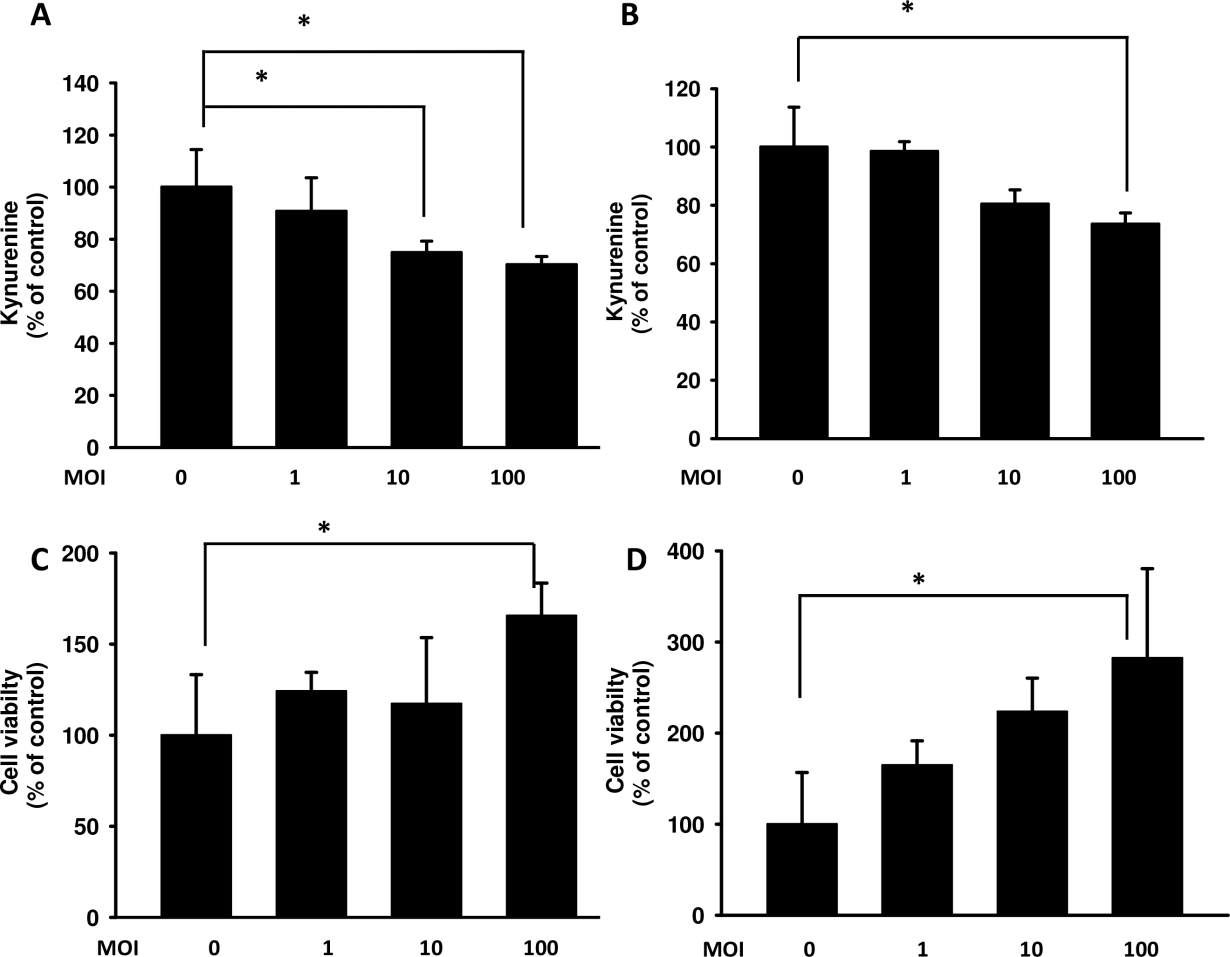
*Salmonella* (S.C.) regulated kynurenine and its impact on T cell survival The B16F10 (**A**) and 4T1 (**B**) cells (10^5^ cells/well) were placed into 6-well plates. After treatment with various MOI of *Salmonella* for 90 min, cells were lysed and Western blot for autophagy marker and IDO were performed. The supernatants of B16F10 (**C**) or 4T1 (**D**) after treated with various multiplicity of infection (MOI) of *Salmonella* were added to Jurkat cells (10^6^) mixed with an equal amount of RPMI medium. After 3 days, cell were harvested and stained with trypan blue. (*n* = 3, data are mean± SD. * *p* < 0.05).

It has been suggested that kynurenine has the ability to increase T cell apoptosis [27]. As shown in Figure 1A and 1B, *Salmonella* can decrease the production of kynurenine in a dose-dependent manner in B16F10 and 4T1 cells. Kynurenine decreased significantly when cells were treated with highest dose of *Salmonella* (multiplicity of infection (MOI) = 100). Thus, we analyze whether *Salmonella*-mediated decrease of kynurenine in B16F10 and 4T1 has impact on T cell survival. The culture medium of cells which have been treated with *Salmonella* was used to incubate Jurkat cells (T cells) to measure T cell viability. The results show that the amounts of T cells are highest when T cells were cultured in medium of cells treated with highest dose of *Salmonella* compared with control group (Figure 1C and 1D). These results suggest that *Salmonella* can increase the survival rate of T cells through inhibiting kynurenine produced by tumor cells.

### *Salmonella* regulated IDO expression through phospho-protein kinase B (P-AKT)/phospho-mammalian targets of the rapamycin (P-mTOR) pathway

**Figure 2:**
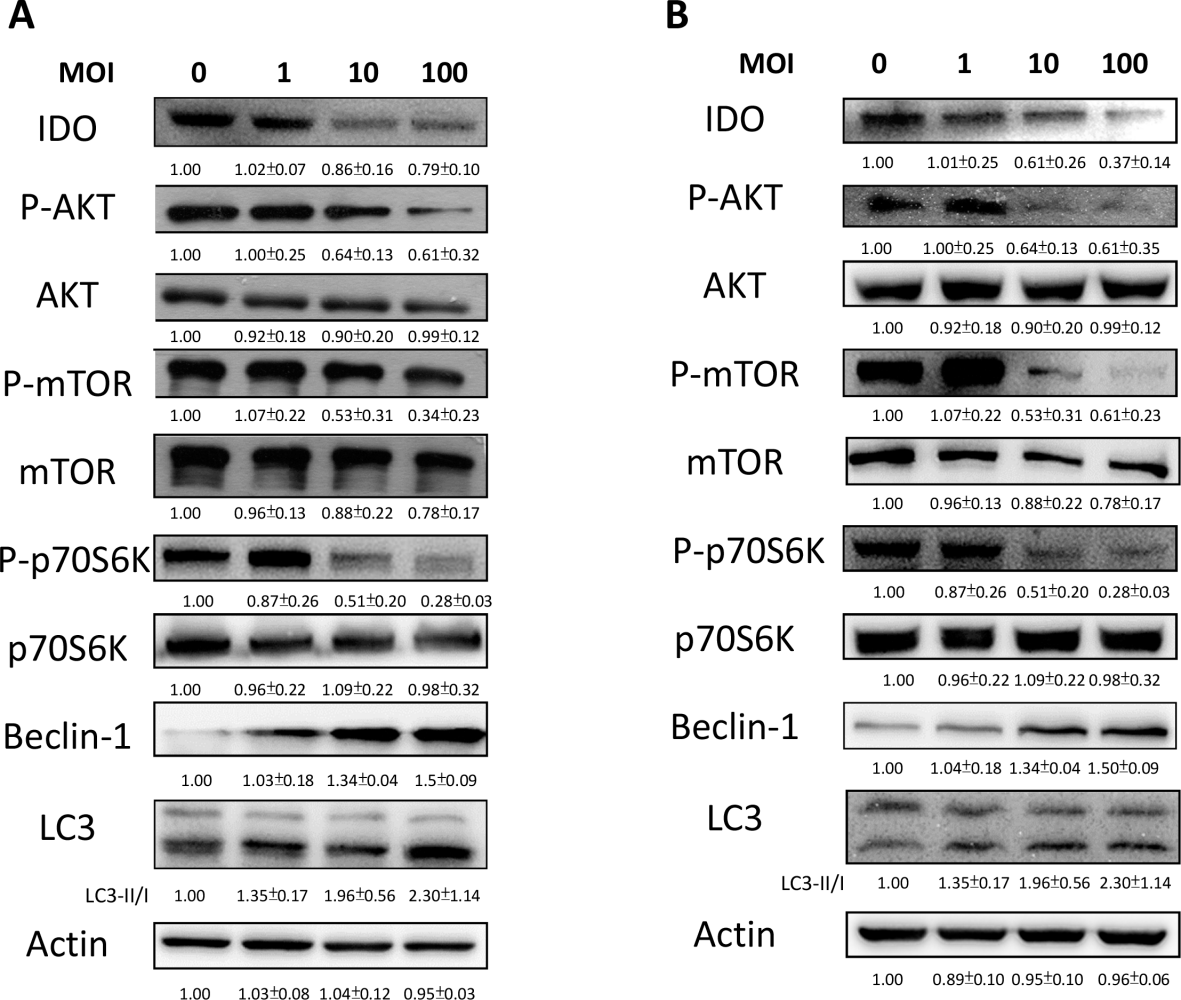
*Salmonella* (S.C.) regulated IDO through the inhibition of an autophagy signaling pathway The B16F10 (**A**) and 4T1 (**B**) cells were placed into 6-well or 12-well plates and then infected with various multiplicity of infection (MOI) of *Salmonella* for 90 min. After 24 hours, cells were lysed and Western blot for IDO, AKT/mTOR/p70S6K and autophagy marker was performed. The inserted values indicate relative protein expression compared to β-actin. This experiment was repeated with similar results.

**Figure 3:**
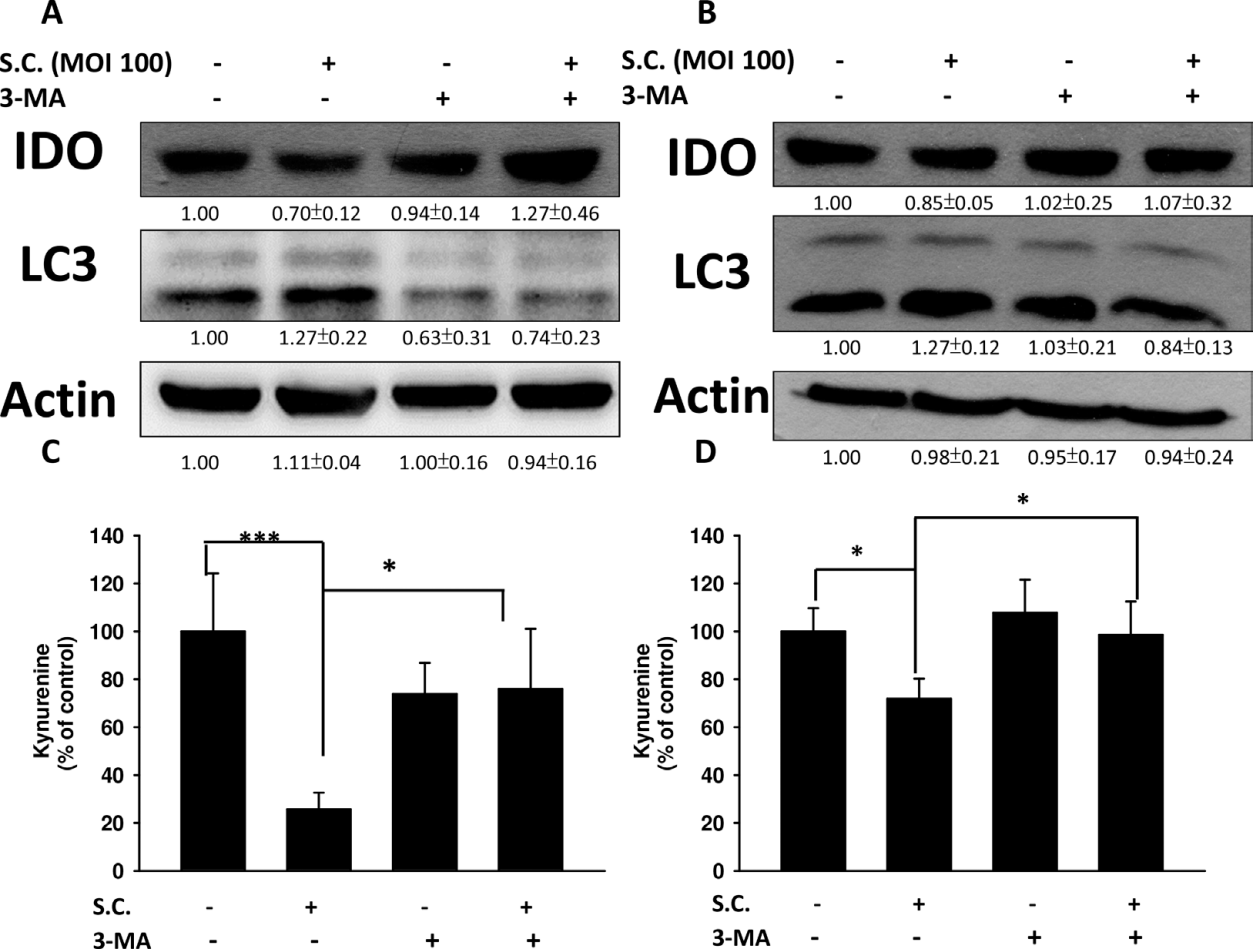
Autophagy inhibitor (3-MA) inhibited the *Salmonella* (S.C.)-induced decrease of IDO The B16F10 (10^5^) (**A**) and 4T1 (10^5^) (**B**) cells were pre-incubated with 3-MA (2.5 mM) for 4 h prior to infection with *Salmonella* (multiplicity of infection (MOI) = 100, 10^7^ CFU/100 μL) for 90 min. The expression of IDO and LC3 protein (A and B) or percentage of control of kynurenine (**C** and **D**) was performed. The inserted values indicate relative protein expression compared to β-actin. This experiment was repeated with similar results. (*n* = 3, mean ± SD. * *p* < 0.05; *** *p* < 0.001).

We demonstrated that *Salmonella* can decrease the production of kynurenine and reverse kynurenine-mediated T cell death. As a result, the enzyme responsible for kynurenine production, IDO, should be inhibited by *Salmonella*. B16F10 cells and 4T1 cells were treated with various degrees of infection (MOI) of *Salmonella* to investigate the ability of *Salmonella* to IDO through induction of AKT/mTOR pathway. The AKT/mTOR/p70 ribosomal s6 kinase (p70S6K) signaling pathway negatively regulates autophagy. We next examined the AKT/mTOR/p70S6K signaling pathway after *Salmonella* infection. In a dose-dependent manner, treatment with *Salmonella* decreased the phosphorylation of AKT, mTOR and p70S6K, indicating inhibition of the AKT/mTOR/p70S6K pathway by *Salmonella* in B16F10 cells (Figure 2A). Furthermore, very similar results were observed when *Salmonella* was used to treat 4T1 cells (Figure 2B). The results demonstrated that *Salmonella* can significantly decrease the expression of IDO and AKT/mTOR signaling both in B16F10 and 4T1 cells. To confirm whether AKT/mTOR signaling pathway truly participates in a *Salmonella*-induced decrease of IDO, cells were treated with autophagy inhibitor (an inhibitor of phosphatidylinositol 3-kinases (PI3K)), 3-Methyladenine (3-MA). 3-MA could restore *Salmonella*-induced downregulation of IDO (Figure 3A and 3B). Determination of the kynurenine expression assay showed that treatment of tumor cells with 3-MA resulted in inhibition of *Salmonella*-reduced kynurenine expression. The production of kynurenine could also be reversed after 3-MA treatment (Figure 3C and 3D). However, adding 3-MA alone had little or even no effect on the expression of IDO and kynurenine, indicating that the autophagy signaling pathway might play the role in the *Salmonella*-mediated decrease of IDO and related catabolic products.

### *Salmonella* reduced the expression of IDO via inhibition of the AKT signaling pathway

**Figure 4:**
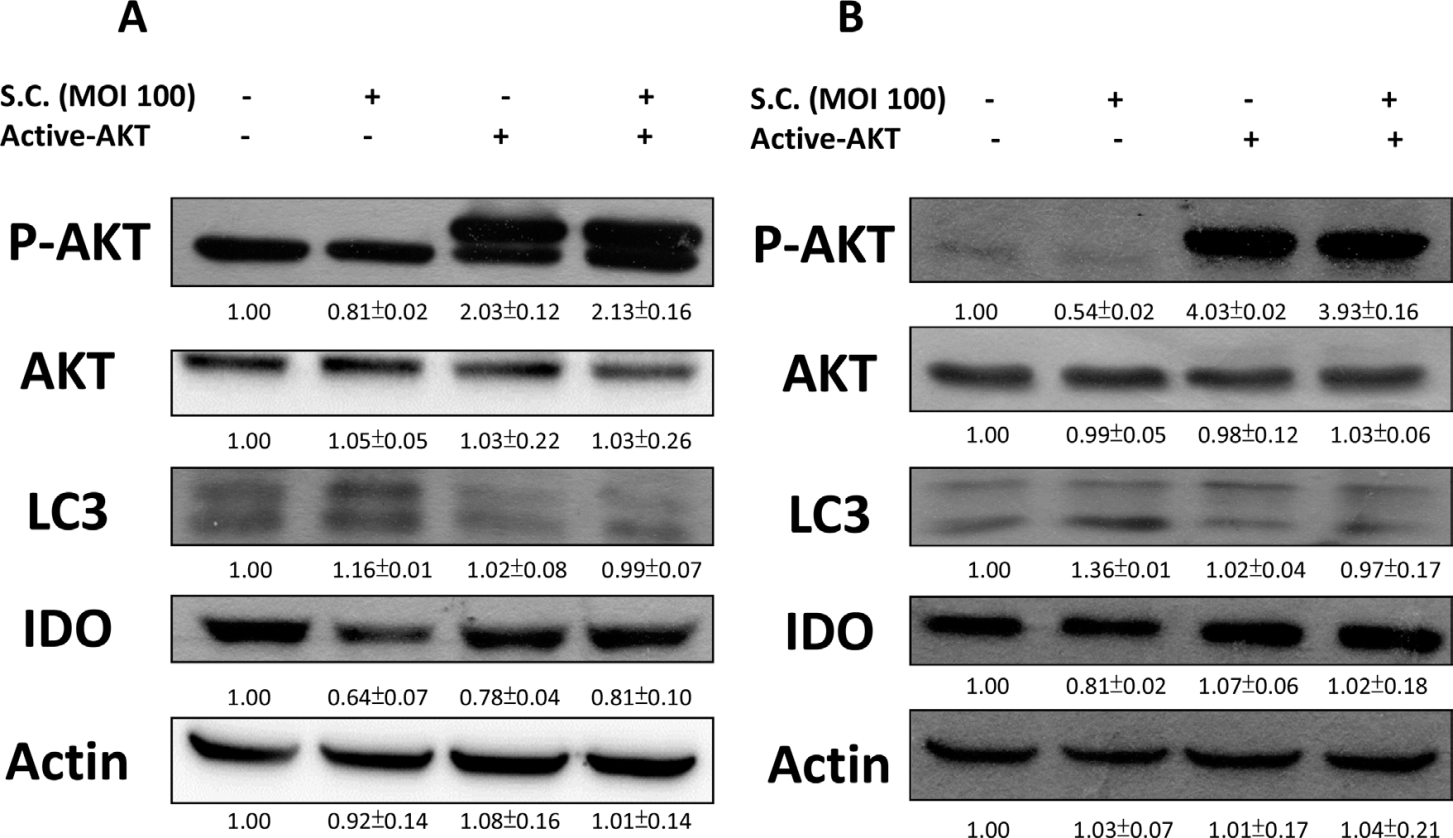
Constitutively active-AKT reduced *Salmonella* (S.C.)-induced decrease of IDO The B16F10 (10^5^) and 4T1 (10^5^) cells were transfected with constitutively active AKT plasmid (5 μg) for 16 hours prior to infected with *Salmonella* (multiplicity of infection (MOI) = 100, 10^7^ CFU/100 μL) for 90 min. The expression of IDO, PAKT, AKT and LC3 protein in B16F10 (**A**) and 4T1 cells (**B**) was determined. The inserted values indicate relative protein expression compared to β-actin. This experiment was repeated with similar results.

The constitutively active AKT was used to confirm our findings. We found that *Salmonella* inhibited the expression of IDO by reducing AKT phosphorylation. The AKT/mTOR/p70S6K signaling pathway was reversed by transfecting constitutively active AKT plasmid. The suppressive effect of *Salmonella* on the AKT/mTOR/p70S6K signaling pathway was relieved by transfecting constitutively active AKT plasmid in B16F10 (Figure 4A) and 4T1 (Figure 4B) cells. By blocking the cascade of autophagy, the expression of IDO in B16F10 cells treated with *Salmonella* and constitutively AKT also reversed, implying that mTOR/AKT participates in the *Salmonella*-regulated decrease of IDO. Transfection of constitutively active AKT plasmid reduced the conversion of LC3-I to LC3-II by *Salmonella* treatment in comparison with vector only control transfection. The *Salmonella*-reduced expression of IDO was also reduced after *Salmonella* treatment by transfecting constitutively active AKT plasmid (Figure 4A and 4B). Our results suggest that inhibition of P-AKT is required for *Salmonella*-reduced expression of IDO in tumor cells.

### *Salmonella* regulated IDO expression *in vivo* and inhibits tumor growth

**Figure 5:**
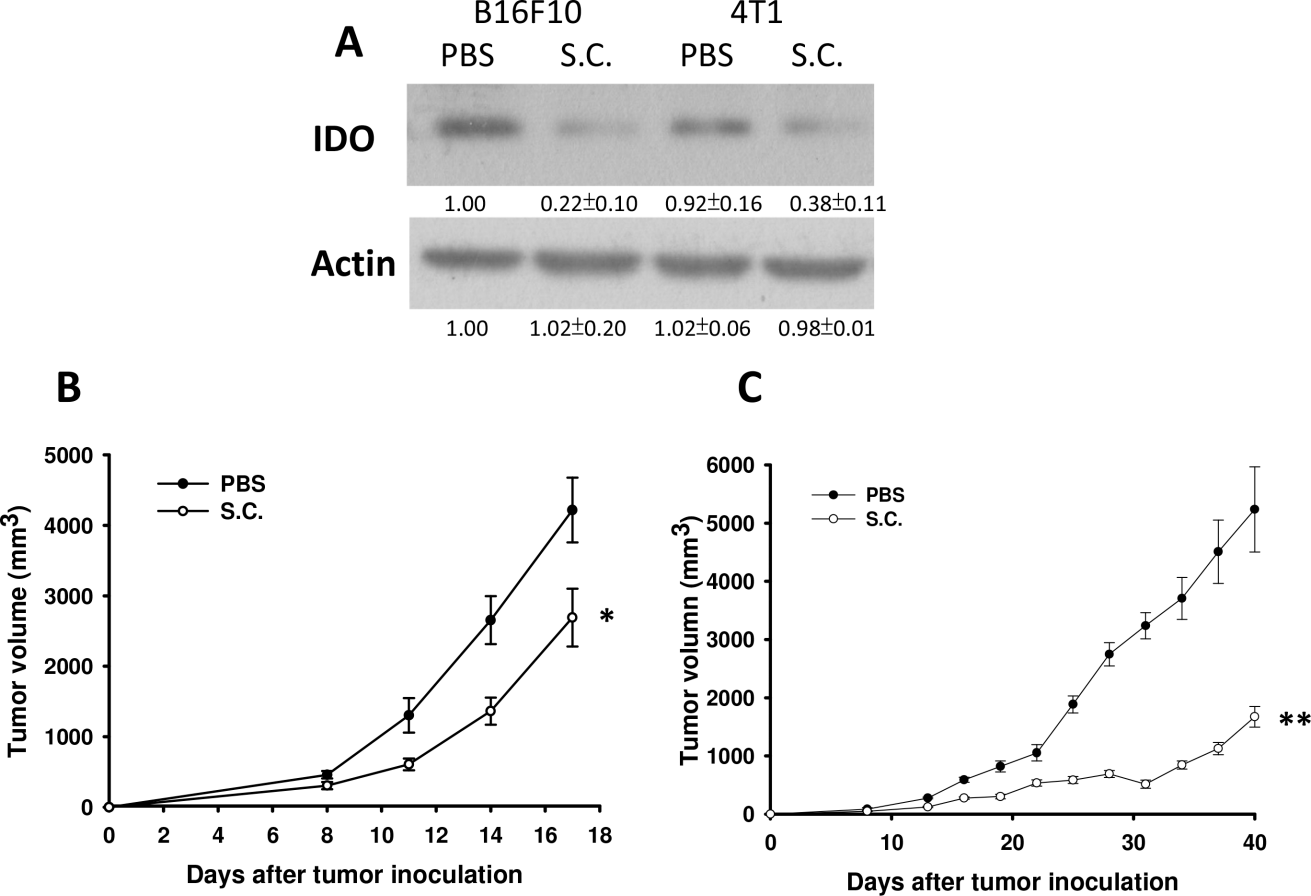
*Salmonella* (S.C.) inhibited tumor growth and downregulated IDO expression *in vivo* Groups of C57BL/6 and BALB/c mice that had been inoculated subcutaneously with B16F10 (10^6^) and 4T1 (10^6^) at day 0 were treated i.p. with *Salmonella* (10^6^ CFU /100 μL) at day 8, respectively. Vehicle control mice were injected with PBS. (**A**) B16F10 and 4T1 tumor tissues were lysed and IDO Western blot analysis was performed on day 3. (*n* = 3, mean ± SD) The inserted values indicate relative protein expression compared to β-actin. This experiment was repeated with similar results. The B16F10 (**B**) and 4T1 (**C**) tumor volumes were measured every 3 days after injection of *Salmonella*. The inserted values indicate relative protein expression compared to β-actin. This experiment was repeated with similar results. (*n* = 10, mean ± SEM. *p < 0.05; **p < 0.01).

**Figure 6:**
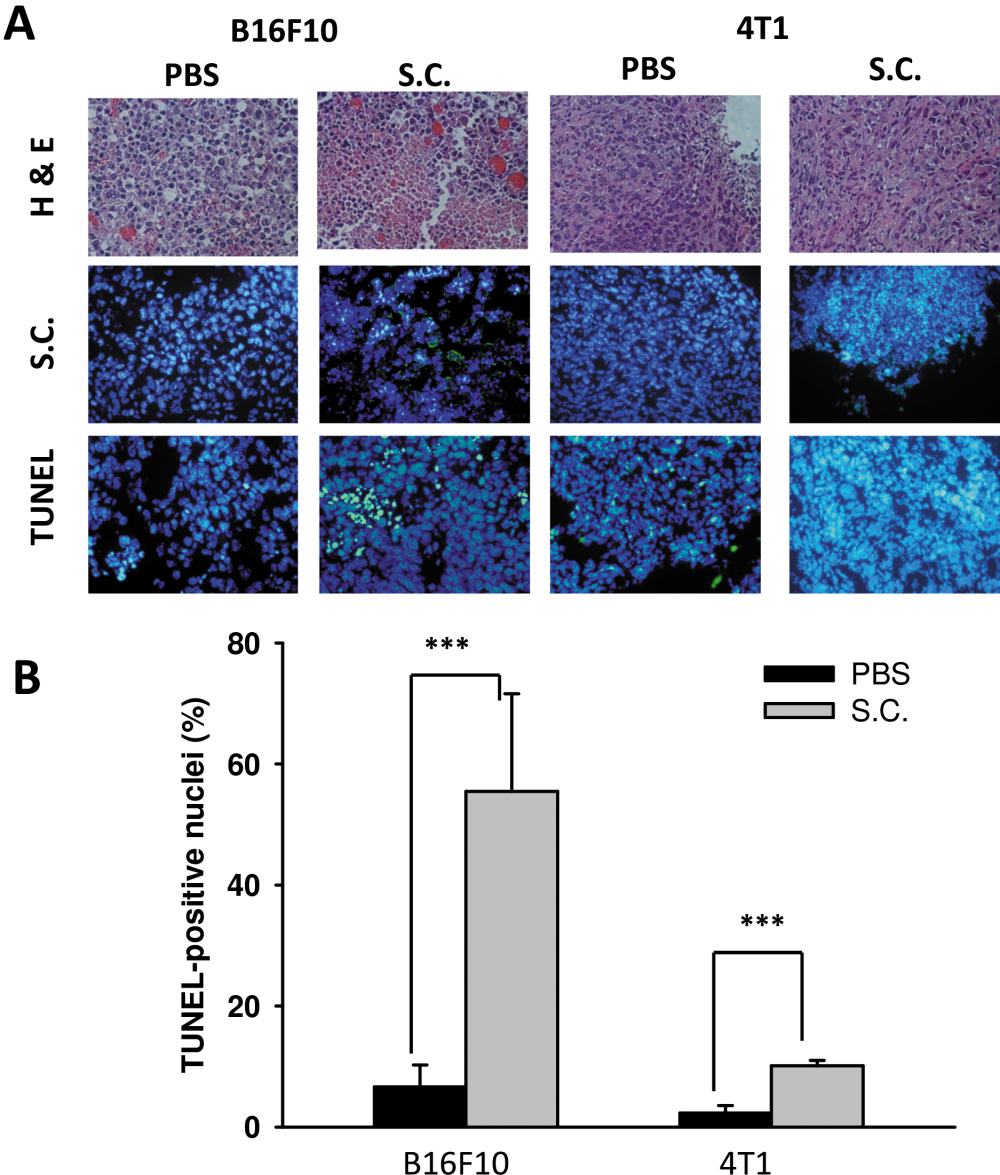
The accumulation of *Salmonella* (S.C.) within tumor region Groups of C57BL/6 and BALB/c mice that had been inoculated subcutaneously with B16F10 (10^6^) and 4T1 (10^6^) at day 0 were injected i.p. with *Salmonella* (10^6^ CFU /200 μL) at day 8, respectively. Vehicle control mice injected with PBS. (**A**) At day 10, tumors were removed for H&E staining and immunohistochemistry staining with anti-*Salmonella* serum to visualize the *Salmonella.* Tumor cells undergo apoptosis in tumor-bearing mice treated with *Salmonella* (S.C.). The C57BL6 and BALB/c mice had been inoculated subcutaneously with B16F10 (10^6^) and 4T1 (10^6^) cells at day 0 were injected i.p. with *Salmonella* (10^6^ CFU /100 μL) at day 8. (A) Tumor tissues were removed at day 10 and TUNEL assay was used to analyze apoptotic cells. (**B**) TUNEL-positive cells were counted from three fields of high-density positive cells in each section to determine the percentage of apoptotic cells. (mean ± SD, *n* = 6. ***p < 0.001).

Tumor cells can create an immune escape microenvironment to facilitate their growth by releasing IDO themselves. Thus, analyzing the IDO expression within a tumor region seems necessary. To verify our findings *in vivo*, tumor-bearing mice were inoculated with *Salmonella* 10^6^colony-forming units (cfu) and sacrificed after 2 days. Tumor tissues were collected and analyzed by immunoblotting assay. The results showed that as the *Salmonella* treatment concentration increased, the expression of IDO decreased (Figure 5A) as previously described. The mean IDO expression for mice in the *Salmonella* treatment group decreased by 54–78% compared with those in the PBS-treated group. *Salmonella* was able to decrease the production of IDO both *in vitro* and *in vivo*. Furthermore, the antitumor effects of *Salmonella* were evaluated in terms of tumor growth of the mice bearing B16F10 or 4T1 tumors. As shown in Figure 5C and 5D, tumor growth was significantly decreased in mice treated with *Salmonella* compared to PBS-treated control mice. The average tumor volume in mice treated with *Salmonella* was lowered by 36% (B16F10) and 68% (4T1) compared with mice treated with PBS. To further examine whether *Salmonella* targeted the tumor sites in mice bearing tumor, we injected *Salmonella* into tumor bearing mice, and observed the bacterial distribution within tumors. As shown in Figure 6A, *Salmonella* predominantly resided in the tumor. The number of apoptotic cells in the *Salmonella*-treated group was significantly higher than in the PBS-treated group (Figure 6B). Taken together, these results reveal that systemic administration of *Salmonella* was capable of inhibiting tumor growth and increasing the tumor cell death.

### Effective T cells infiltration increased due to administration of *Salmonella*

**Figure 7:**
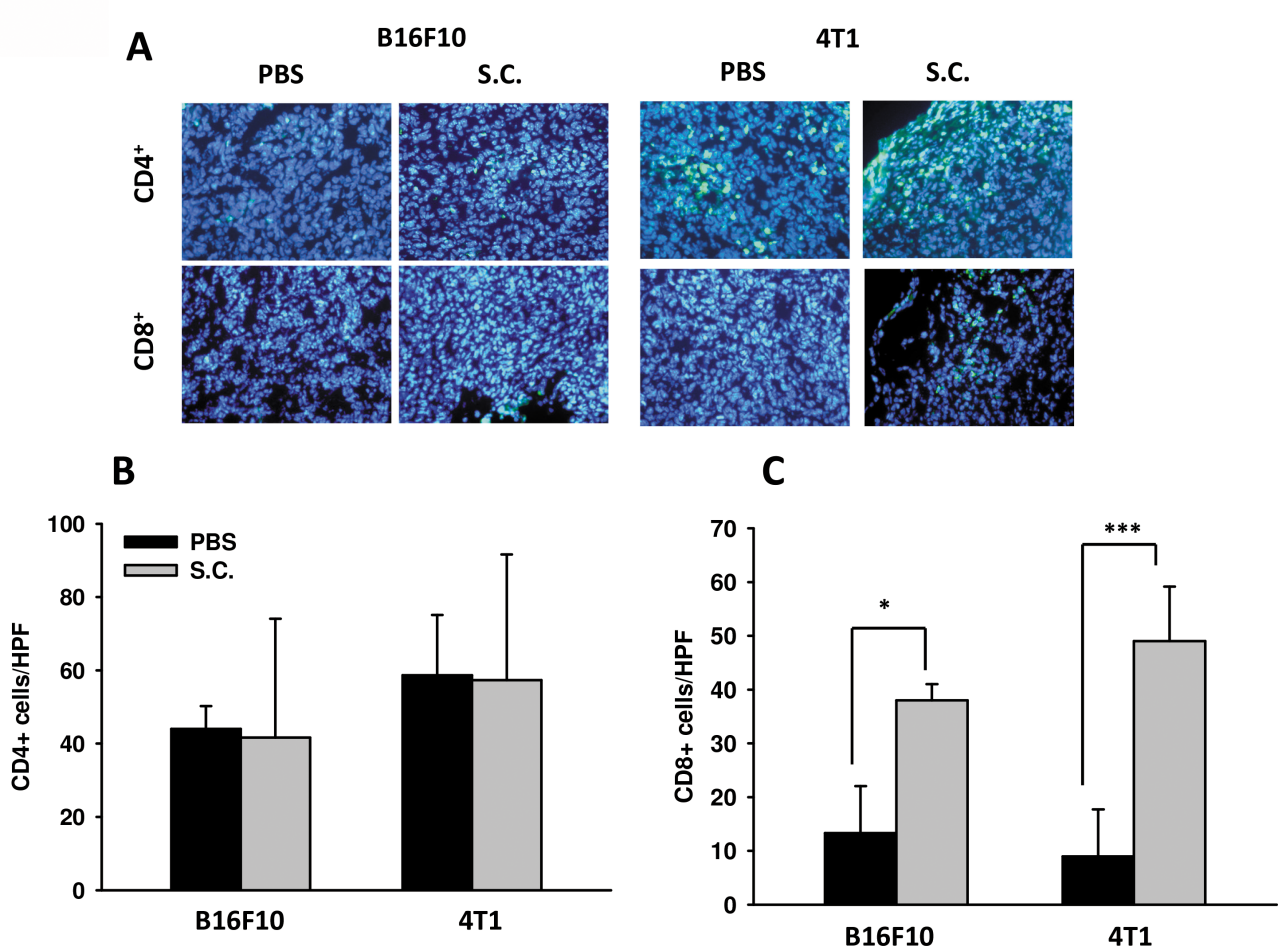
Increases in T-cell infiltrates in the tumors from tumor-bearing mice treated with *Salmonella* (S.C.) (**A**) The expression of CD4^+^-and CD8^+^ positive cells in B16F10 and 4T1 tumor-bearing mice treated with *Salmonella.* The mice had been inoculated subcutaneously with B16F10 (10^6^) and 4T1 (10^6^) cells at day 0 and treated i.p. with *Salmonella* (10^6^CFU /100 μL) at day 8. Tumor tissues were excised after 2 days and immunofluorescence staining was performed with anti-CD4 and anti-CD8. (**B**) CD4^+^ and (**C**) CD8^+^ T cells that infiltrated tumors were determined by averaging the cell numbers from three fields of the highest positively stained cell density at x200 magnification in each section. Each experiment was repeated three times with similar results. (*n* = 3, mean ± SEM. *p < 0.05; ***p < 0.001).

The IDO-mediated tryptophan metabolic product kynurenine has a huge impact on T cells, leading to their inactivation, the inhibition of differentiation to effective cells and even apoptosis. Figure 1C and 1D indicate that *Salmonella* increased the likelihood of T cell survival through downregulating kynurenine production *in vitro*. The ability of IDO to induce T cell apoptosis has been shown. Previous studies have also suggested that *Salmonella* increases the infiltration of immune cells and decreases the amount of IDO, leading to T cell survival both *in vitro* and *in vivo*. Hence, the relation between IDO and the infiltration of CD4^+^ T cells and CD8^+^ T cells was analyzed. The infiltration ofCD4^+^ T cells and CD8^+^ T cells within the tumor region from B16F10 and 4T1 tumor-bearing mice were analyzed by immunofluorescence staining after treatment with PBS or *Salmonella*. The results of immunofluorescence staining are shown in Figure 7A. A notable increase of CD8^+^ T-cell infiltrates in the tumors was observed in *Salmonella*–treated mice (Figure 7B). As shown in Figure 7B, the CD4^+^ T cell amount was not significantly higher in *Salmonella*–treated mice than in PBS-treated mice in both strains of mice. Taken together, these results indicate that *Salmonella* increased infiltrating immune cells and cell death in the tumors.

## DISCUSSION

Immune tolerance has been a hall mark of cancer and one of major barriers developing efficient anticancer treatments. To overcome this hindrance, an effective strategy to evoke host immunity against tumor cells is necessary. Bacteria have a proven ability to inhibit tumor growth; however, the detailed mechanism for this still requires further investigation. Previous studies have suggested that as a pathogen, *Salmonella* not only summons host immune surveillance but can also quickly accumulate within the tumor region, even leading to tumor cell death [7]. Because of its strong properties of activating immunity and killing cancer cells, we believe that *Salmonella* are worth studying and applying to anticancer treatments. The safety concerns for using bacteria can be easily solved thanks to gene modification or antibodies [28, 29]. However, this strain might exhibit lower ability of targeting and killing tumor cells when applied to clinical trial [30]. Researchers constructed an amino acid auxotrophic strain of *Salmonella* and found increased targeting and killing capacity with less toxicity and distribution comparing to original strain [31–40]. Moreover, the auxotrophic strain can be effectively utilized as a treatment targeting several types of cancer or metastatic region [43], and exhibit superior ability of reducing tumor volume comparing to standard chemotherapy [44, 45]. These researches revealed a great promising way using genetically modified bacteria on cancer therapy.

Autophagy is a way in which cells maintain homeostasis and is considered an antitumor target [46], especially the AKT/mTOR pathway, one of the well-studied and targeted autophagy signaling pathways [47]. As mentioned previously, our studies have demonstrated that *Salmonella* can cause autophagy of tumor cells, ultimately leading to tumor cell death [22, 25]. Moreover, evidence shows that autophagy might participate in immune regulation [26]. Hence, we postulate that there are connections between *Salmonell*a, autophagy and host immunity. We observed that the expression of phosphorylated AKT, mTOR and p70S6K decreased in *Salmonella*-infected tumor cells comparing to control groups (Figure 2A and 2B). The relation between autophagy and IDO was verified by using autophagy inhibitor and constitutively-AKT plasmid. The inhibitor and plasmid did not or slightly affect the expression of IDO and kynurenine, but reverse the decrease in IDO and kynurenine when cells were co-treated with *Salmonella* (Figures 3 and 4), which indicates that AKT/mTOR pathway might participate in *Salmonella*-mediated IDO expression. Interestingly, another study inferred that IDO can inhibit tryptophan-sufficiency signals triggering autophagy through the mTOR pathway [48]. *Salmonella* invasion results in the rapid induction of an acute state of cytosolic amino acid (AA) starvation, provoked by host membrane damage. *Salmonella*-induced AA starvation, in turn, down-regulates AKT/mTOR signaling while triggering autophagy and the integrated stress response pathway [49]. *Salmonell*a altered mTOR activity, thereby impacting on host defense pathway. The AKT/mTOR signaling pathways were found to be altered during *Salmonella* infection [50]. Recently, we indeed demonstrated that *Salmonella* inhibit the expression of IDO in colorectal cancer [51]. There are some connection between AKT/mTOR pathway and IDO. The underlying and detail mechanism is worth studying.

We demonstrated that *Salmonella* can inhibit tumor growth in tumor-bearing mice (Figure 5C and 5D), and we investigated the possible mechanism causing the regression. *Salmonella*-induced decreases of IDO were seen both *in vitro* and *in vivo*. Figures 5A and 5B reveal that the expression of IDO within tumor regions can be downregulated by *Salmonella*. We verified these findings through analysis of CD4 and CD8 positive cells. Groups treated with *Salmonella* had a higher infiltration of CD8 positive cells compared to controls (Figure 7). This might be one of the explanations for why immune cells infiltrate the tumor region after treatment with *Salmonella*. *Salmonella* not only kills tumor cells by its bacterial toxicity but also recruits and even effectively activates host immune cells to attack; this might be a good topic of future study or might help in developing supporting treatments for patients who have impaired immunity. *Salmonella*-reduced IDO expression is not solely responsible for tumor immune tolerance. The tumor microenvironment contains immunosuppressive factors such as arginase, vascular endothelial growth factor and TGF-β. Recently, we also observed that *Salmonella* could inhibit the expression of programmed cell death protein 1 ligand 1 (PDL1) and programmed cell death protein 1 ligand 2 (PDL2) in melanoma cells. There are many other mechanisms by which the antitumor T cell response is inhibited. Herein, we provided one of possible mechanisms for *Salmonella* antitumor activity. Once successfully evoked and combined with other treatments, future *Salmonella*-related treatments could shorten the treatment time and reduce side effects. The bacteria strain employed here has been utilized as a vector delivering DNA into mice [52]. Using *Salmonella* combined with drugs capable of reducing IDO and inhibiting tumor growth may be other choice for immunotherapy one day. In future studies, a further analysis of immune-related cytokines is needed, which might provide a reference for monitoring tumors or treatment progress after treatment with bacteria. In addition, because we demonstrated that *Salmonella* can break tumor immune tolerance, this might be another aspect to consider for antitumor treatments, even the application of bacteria to immunotherapy.

## MATERIALS AND METHODS

### Bacteria, cell line, plasmid, reagents and mice

Vaccine strain *Salmonella enterica* serovar *choleraesuis* (*S*. Choleraesuis; S.C.) (ATCC 15480) was obtained from Bioresources Collection and Research Center (Hsinchu, Taiwan) [53]. Bacteria were maintained in L.B. plate and propagate in L.B. broth for using. B16F10 (mouse melanoma) [53] and 4T1 (mouse breast cancer) [54] cells were maintained in 10 cm Culture dish with Dulbecco's Modified Eagle Medium containing 1% Penicillin-Streptomycin (100 units/mL penicillin and 100 μg/mL streptomycin), 2mM l-glutamine and 10% fetal bovine serum. Jurkat cell line (human T lymphocyte; (ATCC TIB-152™)) was a kind gift from Professor Hui-Chen Chen (China Medical University) and maintained in HyClone RPMI 1640 medium containing 10% FBS. All cells were passaged every two to three days and incubated at 37°C, 5% CO_2_. Autophagy inhibitor (3-Methladenine; 3-MA) were purchased from Merk (Darmstadt, Germany). Constitutively active AKT plasmid was kindly provided by Dr. Chiau-Yuang Tsai (Department of molecular immunology, Osaka University) [55]. Cells were treated with inhibitors for 4 h or plasmids for 16 h in serum free medium prior to adding *Salmonella* (MOI = 100) into cells for 90 min at 37°C, 5% CO_2_. C57BL/6 (B16F10) and BALB/c (4T1) mice were purchased from National Laboratory Animal Center of Taiwan. The animals were maintained in a pathogen-free animal care facility in isothermal conditions with regular photoperiods. The experimental protocol adhered to the rules of the Animal Protection Act of Taiwan and was approved by the Laboratory Animal Care and Use Committee of the China Medical University (permit number: 101–20–N).

### Western blot analysis

The protein content in each sample was determined by bicinchoninic acid (BCA) protein assay (Pierce Biotechnology, Rockford, IL). Quantified each sample concentration to 60–80 μg and add 4 × SDS sample dye and then denatured sample for 10 min at 95°C. Proteins were fractionated on SDS-PAGE, transferred onto Hybond enhanced chemiluminescence nitrocellulose membranes (Amersham, Little Chalfont, UK) and detected with antibodies against IDO (Thermo Scientific, Rockford, IL), the mammalian target of rapamycin (mTOR) (Cell Signaling, Danvers, MA), phosphor-mTOR (Cell Signaling), protein kinase B (AKT) (Santa Cruz Biotechnology, Inc. Santa Cruz, CA), phosphor-AKT (Santa Cruz Biotechnology, Inc.), p70S6K (Cell Signaling), phosphor-p70S6K (Cell Signaling), microtubule associated protein 1 light chain 3 (LC3) (Novus Biologicals, Littleton, CO), Beclin (Novus) and β-actin (Sigma Aldrich). Rabbit anti-mouse IgG-peroxidase antibody (Sigma Aldrich) and goat anti-rabbit IgG-peroxidase antibody (Sigma Aldrich) were used as the secondary antibody and protein-antibody complexes were visualized by enhanced chemiluminescence system (Amersham) [56]. The signals were quantified with ImageJ software (rsbweb.nih.gov/ij) [57].

### IDO functional assay

B16F10 or 4T1 cells were plated in 12 well culture plates for 24 h, and treated with different MOI of *Salmonella* for 1.5 h. After 24 h, cells were collected and centrifuged at 12,000 rpm at 4°C, 10 min. The supernatants were then heated at 60°C, 15 min. 100 μl supernatants were added equal amount of IDO assay buffer (K_2_HPO_4_ 50 mM (Sigma Aldrich), ascorbic acid 20 mM (Sigma Aldrich), methyl blue 10 μM (Sigma Aldrich), catalase 100 μg/ml (Sigma Aldrich) and harvested at 37°C, 10 min. 40 μl of 30% TCA (Sigma Aldrich) were added and centrifuged at 3000 g, 15 min. Afterward, the 125 μl of supernatants were mixed in 125 μl of 2% Erchlich’ s reagent (Sigma Aldrich) in acetic acid in 96-well plates. The absorbances of kynurenine were detected by spectrophotometer at a wavelength of A_490_. The data were performed as percentage of control. All of the IDO functional assays were done in triplicate.

### Animal studies

Groups of 10 C57BL/6 mice or BALB/c mice were inoculated with B16F10 or 4T1 (10^6^) cells, respectively. After 7–9 days when the tumors were about 50–100 mm^3^, *Salmonella* (10^6^ cfu) or PBS was injected intraperitoneally (i.p.) into mice bearing either B16F10 or 4T1 tumor. Tumor tissues were collected after 3 days and prepared for western blot. To analyze tumor volumes, tumors were measured every 3 days in two perpendicular axes using a tissue caliper and the tumor volumes were calculated as (length of tumor) × (width of tumor)^2^ × 0.45. For immunoblotting assay, TUNEL assay and immunofluorescence staining, the tumor samples were collected on day 10.

### TUNEL assay and immunofluorescence staining

Cryostat sections (5mm) were prepared and fixed. Tumor tissues were then processed in 5-μm sections and stained with hematoxylin/eosin (H&E). The accumulation of *Salmonella* within tumor region was described in the previous study [57]. Cell apoptosis within tumor region was analyzed by TUNEL assay according to the manufacturer's protocol (Promega, Madison, WI). TUNEL-positive cells were counted under the microscope in high-power field (HPF). The apoptosis index was determined by the percentage of TUNEL-positive among total cells of each sample [8]. To analyze the expression of CD4 or CD8 positive cells within tumor region, tumor cryostat sections were incubated with antibodies against CD4 (BD Biosciences, San Diego, CA) and CD8 (BD Biosciences). Slides were then incubated with fluorescein (FITC) anti-rat IgG (H+L) for secondary antibody and counter stained with 4′,6-diamidino-2-phenylindole (DAPI). The infiltrating cells were quantified by averaging the number of each cell type in three areas of highest cell density at x400 magnification in each section [8].

### Cell viability assay

Tumor cells were plated in 6 well culture plates and treated with various MOI of *Salmonella* for 1.5 h after cells have been seeded for 24 h. The supernatants of *Salmonella*-treated B16F10 or 4T1 were added to Jurkat cells mixed with equal amount of RPMI medium. After 3 days, cell survival was assessed using the trypan blue exclusion assay [25]. All of the IDO apoptosis assays were done in triplicate.

### Statistical analysis

The unpaired, two-tailed Student *t* test was used to determine the differences between groups for comparisons of kynurenine, tumor volume, tumor weights, T cell death. The *p* value less than 0.05 is regarded statistically significant.
